# ‘A German Whore and no Money at that’: Insanity and the Moral and Political Economies of German South West Africa

**DOI:** 10.1007/s11013-019-09663-4

**Published:** 2019-11-18

**Authors:** Mattia Fumanti

**Affiliations:** Department of Social Anthropology, The University of St Andrews, 71 North Street, KY16 9AL St Andrews, Fife, UK

**Keywords:** Mental illness, Narratives, Feminist ethics, Gender, Moral and political economies, German South West Africa

## Abstract

While the links between colonial psychiatry and racism figure prominently in histories of the diagnosis, treatment and institutionalisation of the mentally ill in Africa, there is an absence of patient-centred accounts, in the analysis of the efforts of the colonial-era subjects themselves to be pro-active not merely as the mentally ill, by clinical or court definition, but as persons embedded in social relationships with their kin and significant others. Moreover, despite an emerging scholarship, little is known of the experience of European settlers. In this respect there is a need for a more balanced representation, one that shows the ambivalence of colonial psychiatry and its reach into the lives of colonial subjects, Africans and Europeans alike. In this paper I focus on the narratives of a settler in German South West Africa and her efforts to escape diagnosis and institutionalisation. In building on a feminist approach to illness narratives, in particular on the idea of bearing empathic witness, I will explore the ways in which illness narratives can reveal the complex moral and political economies of the colonial world.

## Introduction

*When we rave, we rave against the whole world, not our family,*

*Gilles Deleuze* (2004)

On April the 4th 1916, a woman named I.L. sent a letter to Arnold Schad, the Mayor of Swakopmund.^1^ This wasn’t the first, nor would be the last of the letters, that I.L. sent to the German administrative authorities in German South West Africa, between January and May 1916. I.L. pleaded desperately with Schad for material assistance and with deportation to Germany,

*I report to the authorities with the intent to be by force removed to Germany*^2^

I.L. arrived in German South West Africa in 1911.^3^ She worked in Windhoek as a shop assistant. Here she met her fiancé, Mr. F, a local butcher. I.L.’s engagement to Mr. F. ended abruptly in the summer of 1915. We do not know exactly the reason, but in her letters I.L. had been labelled a prostitute and a lunatic. Her letters, I argue, are a desperate plea to be heard. The letters are a forum to speak and resist the dehumanising process of exclusion that followed the break-up with her fiancé. In a dramatic crescendo of anger, rage and despair, I.L.’s voice can be heard loudly as a rave against her ex-fiancé, the German colonial authorities, the colonial world, and even against the First World War. I.L.’s letters illustrate much more than her life and her supposed insanity. They are, I argue here, revealing of the place of women and insanity within the moral and political economies of German South West Africa. These letters, I propose, bring forward a fresh insight into the complex interrelationship between madness, class, gender and race in the life of German colonial settlers and more widely of the colonial world.

Links between western biomedicine, colonial psychiatry, racism, and oppression figure prominently in histories of the diagnosis, treatment and institutionalisation of the mentally ill in Africa (Deacon 1996; Jackson 2005; McCulloch 1995; Swartz 1995a, b). The colonial asylum provisions for black patients were poor (Heaton 2013); treatment regimens were discriminatory as were scientific justifications for diagnosis on the basis of presumed biological differences (Keller 2001). It follows, some historians argue, that the very diagnosis of African patients as insane served racial theories, constructing the colonial world as orderly and sane (Vaughan 1993). Further, committing the ‘mad’ to an asylum was often actually the way to remove from society ‘troublesome’ Africans, who disturbed discipline in farms and mines, or who threatened the social peace and decorum. Troubled and troubling, they were more likely to be diagnosed and institutionalised (Edgar and Sapire 2000; Sadowski 1999, 2004; Vaughan 1983, 1991). Where the histories largely fall short is in patient-centred accounts, in the analysis of the efforts of the colonial-era subjects themselves to be pro-active not merely as ‘the mentally ill’, by clinical or court definition, but as persons embedded in social relationships with their kin and significant others.^4^ Moreover there is an absence, only partly filled by emerging scholarship, on the experience of European settlers in the African colonies (Jackson 2013; Parle 2007; Wilbraham 2014).^5^

In addressing these gaps, my aim in this paper is to explore the structures, strictures and contradictions that underpin the relationship between colonialism, power, psychiatry and western biomedicine (Vecchio-Good et al. 2008). As Vaughan (1983, 1991) has pointed out for colonial Africa, biomedicine was an important aspect of the colonial project used to impart control of the colonial subjects and their bodies. However more often than not the reach of biomedicine, and in this case psychiatry, on the colonial subjects was limited and ineffective. Paradoxically, as I argue here, while Africans continued to engage with plural contexts of healing, it is European settlers, often ‘vulnerable’ and ‘undesirable’ subjects, that were more likely to encounter medicalisation and institutionalisation. This is particularly so for colonial South West Africa, both under German and South African rule, where the colonial authorities actively pursued a policy of expulsion of the mentally ill. My focus on I.L.’s illness narrative will bring to bear the vulnerabilities of European settlers’ *vis*-*à*-*vis* early colonial psychiatric interventions and bio-medical discourses and practices, while revealing important aspects of the colonial world.

In this paper I begin with the premise that narration and representation are important ethical concerns that require a critical ethical approach. This is because any narrative approach needs to rest on the premise that autobiographical power is central to a person’s sense of empowerment as it allows redressing the silencing and dehumanising process of diagnosis and medicalization (Myers and Ziv 2016). The power over self-narrative reveals personal efforts to escape appropriation and silencing of a patient’s narratives. Without this premise, any narrative approach would risk adding further injuries and reproducing the person’s sense of social defeat, as the repeated instances of physical or social insults in which one party is made to feel less powerful than the other (Lovell 1997; Kleinmann 1988).

Here, my argument builds on feminist historians’ contributions situated at the intersection between ethics and narrative approaches. This is an approach that takes the practices of storytelling, listening and bearing careful witness to personal stories, not simply to understand the unique circumstances of individual lives, but also on the relational quality of individual narratives and their capacity to reveal subjectivities in transformation and in relation to significant others, with kin and more widely with society and the world (Tamboukou 2010). As Parry underlines (2015), this is the basis of feminist ethics, one that focuses on the individual, on experiences, on intimacy, on morality and positionality and which rejects universalisms. These, as Parry and others have argued (Zemon Davis 1987, 1995), have often silenced women, and other marginal groups, people of colour, the disabled, and the mentally ill. On the contrary an ethical feminist approach would help recall these lost voices. By refusing to focus on wider universalists claim to the truth, but instead in paying special attention to the narratives in themselves, their capacity to draw from personal relations, but also to draw connections with the wider moral and political economies that support them, these accounts will encourage us to reject totalizing and universalist approaches to illness narratives in favour of those that offer more nuanced understandings of the positionality of the individuals at the heart of them.

This is an issue that is particularly central to the representation of mental illness narratives which appear from outside as fragmented/incoherent and fictional (Lovell 1997). I.L.’s letters belong to these kind of narratives. They appear fragmented/incoherent and at times fictional. This is true both of I.L’s personal account but also of the archival material, with its silences and inevitable absences. In this sense an approach to illness narratives requires an effort, as Lovell puts it,

To join at least two concerns: the aesthetics of storytelling, with its metaphoric quality and capacity to evoke real and imagined worlds through sensory images and sensate feeling, and the analytic but dynamic treatment of social action as text. (1997:355).

For this reason I argue, a feminist ethical approach is opened to the possibility of multiplicities (Tamboukou 2010), even for the recognition of the space that imagination and fiction have in the crafting of a narrative. As Zemon Davis underlines (1987), fiction, from the latin *fingere*, does not entail a fictional account, but the process of forming, the shaping and moulding of different elements in an account towards the crafting of a narrative. Davis invites us to look at the ways in which these stories were told rather than to their structure. In writing on the letters of remission of women standing trial in XVI century Europe, Davis argues that these letters appear in the archives as a mix of judicial supplications, both historical accounts and stories (1987:4). I.L’s writings are both a story and an historical account. Hers is a story of violence and exclusion, as well as an historical account of the moral and political economies of German South West Africa. And because this is both a story and an historical account, one needs to pay attention to its absences and silences, as well as to its imaginative. To the capacity that such narratives have in drawing from imagination, and to make others draw from it. In this sense, imagining and making others bear witness, is one way in which people diagnosed with mental illness can make others recognise their shared humanity, by placing one’s experience within the patient’s understanding of humanity and well-being (Fumanti 2018). In this sense one cannot begin to understand I.L’s story simply in terms of mental illness, unless we understand first what it meant for the protagonist to be human.

In bearing empathic witness to I.L’s account, I aim to show how I.L.’s writings reveal her effort to remain human amidst the brutality of the colonial world.

## I.L.’s Biographical Notes

I.L.’s biography is extremely fragmented. I.L. was born on August the 2nd 1880 in a village in Silesia, near Breslau, (now Wroclaw, Poland). In the late nineteenth century Silesia had a prevalence of German speaking people and its economy was based on coal mining, agriculture and heavy industry (Schofer 1975).

I.L. came from a German-speaking working class family. Her father was a miner and her mother a housewife.^6^ We do not have other information about her life. In October 1910 she responded to a job advert and her application was processed through the office of the *Frauenbund der Deutschen Kolonialgesellschaft* (The Women’s Association of the German Colonial Society). Founded in 1907 by aristocratic and upper-class German women, the association promoted the immigration of women, mostly working class, to the colonies, for work and to preserve German identity and racial purity (Bechhaus-Gerst and Leutner 2009). We know that I.L. arrived in German South West Africa in September 1911 and worked in Windhoek for Mr. Hermann Liwinowsky. Liwinowksy, who arrived from Lozen in East-Prussia in the early 1900s, was running a successful drapery store on KaiserStraße in Windhoek (Wolfsohn 2017).^7^ I.L. worked there as a shop assistant. I.L. and lived in Klein-Windhoek, in the *Genossenschaft der Oblaten der Heiligen und Unbefleckten Jungfrau Maria* (Cooperative of the Order of the Oblates of the Saint and Immaculate Virgin Mary) (Fitzner 2012:72).

I.L. travelled to Germany in 1914 and returned a few months later.^8^ We can assume that she travelled to Germany to see her family and announce her engagement. There is mention of a dowry and of a brother living in Karibib, a small town North of Windhoek, but no records of his presence can be found. She also mentions another brother in Tenerife and a sister that lived in the Canary Islands or Cape Town.

Sometime in 1915, her fiance’ broke off the engagement and evicted her from his house. I.L. was homeless and was not allowed to live in the *Genossenschaft.* For a few months, until December 1915, she stayed in different Windhoek’s hotels. In January 1916 she moved to Swakopmund and lodged at the Hotel Eggers. It is at this point that she began her plea for immediate repatriation. However, her already precarious position had been further constrained by the war. The South African army had occupied German South West Africa in July 1915 and imposed martial law (Hartmann, Sylvester and Wallace 1998).

I.L. was now alone, in an occupied country. She was unemployed, living in hotels and in receipt, as an indigent, of war rations.^9^ Her ex-fiancé had left her without any financial assistance. Furthermore, because of the war, the Wormann Linie had suspended all travels between Germany and its colonies.^10^ I.L. was now trapped in occupied German South West Africa. Feeling desperate and in dire straits she began writing letters to the German administrative authorities, to the mayors of Windhoek and Swakopmund, and to other prominent members of the colonial society.

In the letters she appears angry, demanding compensation. She curses her ex-fiancé, hoping for a violent retribution. Her sentences are often truncated. There are exclamation marks and spaces left intentionally blank. She uses profanities. Her handwriting is unclear. But, most importantly, she wants to be heard. She resist being silenced. She wants to set the record straight and rehabilitate her name. She feels she has been the victim of a conspiracy. This, she argues, is the consequence of gossip and rumours. These letters are I.L’s ‘*J’accuse’* against certain individuals, and more widely against the colonial society. I.L. accuses the police of corruption and sexual harassment. She denounces the double moral standards of the colonial world. She refutes the chauvinistic language men have used to speak to her and of her.

In the next section I reproduce large sections of two of I.L.’s letters. In bearing empathic witness to her story and hope to restore her voice and give her the space to be heard once more.^11^ Her words, I argue, will reveal much of the way in which class, gender and sexuality intersected in German South West Africa, shaping the lives of Europeans and Africans alike.

## Bearing Empathic Witness in the Archives. Restoring I.L.’s Silenced Voice

*Windhoek, 2. Jan 1916*

*To The German*-*Imperial Government to the attention of Mr. Governor Kurt Kastel,*^12^

*Due to the ‘dirty things’ which continue to be attributed to me, I would like to leave Windhoek. Mr Uhlemann, has given me the attached hotel bill.*^13^*I do not have means to pay. These should be paid by butcher F. Regarding this issue, I already spoke to Dr. Buergermeister and Dr. Stark,*^14^*but also with no results. If I can’t get my support, I no longer know what to do. I need laundry and clothes* etc*. From the statements I made to the municipality it comes to light that this is all provoked to make D.S.W (Deutsch Südwestafrika) impossible for me.*

*I should work as a prostitute on the street to____ [implied here is the verb “earn”] the money for my return journey. ‘German whore and no money at that’, that is what they always said of me.*

*At Uhlemann it was said, “We need a monkey in this time, the old box [pejorative for an old, useless woman] is just good enough”. In another occasion they said, “We can do it with her (sexual. connotation), she is hussy like her brother.”*

*What is the meaning of all the mock and ridicule directed towards my family in the colony, I have not been able to make sense of for the past one*-*and*-*half years.*

*He should, through perverse and other activities be completely wrecked. He should be blind, walk on crutches.*

*We should become old and grey before finding our kin. My brother and I are being deported.*

*Without a ring I am not allowed to enter the cooperative. It is said that E.J. has one already, and Hoff has one as well* - *that is one from F. and the other one was mine. It is said that they were acquired through dirty means. How have these other ‘women’ acquired their rings?*

*I have been asked to work, others do not need to work and still get a salary.*

*When he travels to Germany, he goes 1st class, but with public girls. He thus has these affairs here, and when he goes to Germany, he also has them publicly over there.*

*Nevertheless if I receive legal compensation this is no longer relevant. And still my kin and I will be harassed and disgraced. Earlier L. and J. were mentioned to me. Today, other women are further brought to my knowledge.*

*Could I perhaps be informed by the Government if my dowry is still here? It is precisely explained how these things look. This was a large travel basket and a box.*

*It is also said that he obtains no reputation from me [potentially referring to not bearing children]. He only needs to give me what I should get. I lived with F., every night I slept with him.*

*With all this I justify my request to leave Windhoek as soon as possible and go to Germany, but accompanied.*

*My sister is supposedly in Cape Town, another time in Las Palmas, my brother is in Tenerife.*

*Of what concern is it to the public at which age I marry?*

*I would like to work to pay my bills. Have I not been in employment not to be dependent on any human being? How was I treated? How does F. treat such women, and how does he treat me? Can the government support me, as my given right? As a married woman shouldn’t I live with the support of a man, as others do? My state of emergency is used to the amusement of various audiences.*

*Yours respectfully,*

*Mrs F.*

*Hotel Hohenzollern,*

*Windhoek*

*Ps: I will be forced to work, I was already told 1 ½ years ago. As what F. says about me when we were married, I was never ill as often as I am now, since I have come back from Germany.*

*Swakopmund, 04/04/1916*

*To the Mayor Mr. Schad,*

*As can be seen from the letter of Mr. Dr Gumprecht my name I. L. was already doubted as in Windhoek, where Mrs. Hoff as I. L. (from the town of H.) is recognized, even as Mrs. F., (Butcher) anyway she was married to Major Franke in a civil marriage, with two men as witnesses.*^15^

*I did not pluck this from the air.*

*Apparently she owns documents in my name (Birth certificate) and is identical to I. L. from S, who was searched and wanted when aged 18. But today this is apparently me, but I am from H.*

*Apparently she also worked in the farms and was employed with my references, also in Swakopmund. I was asked previously in Windhoek if I had worked in employment in Swakopmund, but I didn’t pay much attention to it. I also apparently participated in cinema movies. But it was Hoff, as I can testify myself, in a movie production at Mr. Leitner. During the show, they screamed, ‘is this not Ms. F?’ I suppose it was butcher S.*

*If I am so alike to Mrs. Hoff, which I doubt, since I am known as Frau F. at the cooperative, I would like to repeat my appeal for immediate departure.*

*She is highly esteemed as a common girl, and how they treat me as the same one, raw and brutally.*

*Butcher F. apparently also no longer recognises me from this mix*-*up, otherwise he wouldn’t have sent the bride on the street as a prostitute, who intentionally came from Germany through the colonial government…*

*That’s because we are from a prostitutes’ family, something that is objectionable for German South West Africa. That is why I should be deported from the country for ‘immoral life change and huge uncleanliness’. If I could leave for Germany of course, in this or that way, I do not care for the opinion of German South West Africa.*

*Instead I should go hand to hand, mainly in the Hotel business through punishable deeds, but I am always this, it is my language to be placed as common.*

*I deliver myself to the authorities with the intent to be ‘by force’ removed to Germany.*

*Butcher B. is right when he tells F. ‘What do you want with the old Plum?’ [Insult meaning old, useless woman]*

*I would thus like to ask Mr. Mayor Schad for my entitled support which is medically approved and is stating that I cannot be employed.*

*Mr Dr Brenner also asked if I contracted any sexually transmitted diseases due to my sexual intercourses with F.*^16^

*I would like to ask again for support, as according to a statement made by Mr. Dr. Stark, F. is liable to pay my support for the time of the war, as he made me come from Germany just for the issue of marriage. Apparently, he can’t be forced without a court order.*

*I, in turn, was forced to do so much through court orders, things which would be punishable under German law.*

*I was raped by a lot of people, as should be clear from the medical certificate, and Mr. Dr Gumprecht’s letter.*

*‘Yes, old screw, necessity breaks iron’, Suggestion for Movem. For ! ____ [intentionally left blank in text, suggesting sexual intercourse] and ‘Why don‘t you look at the street’*

*I should return to Germany, where the leftovers come from, (‘at least we have been proudly presented as children and with fat cheeks’).*

*Recently nevertheless, I must have had hallucinations after talking to the police in Windhoek. And would this policeman get away with what he said to me? ‘If you have no means to survive, then why don’t you put yourself here?’ and hit himself on his ass while saying this.*

*How should I make sense of this?*

*May I, through Mr. Mayor, perhaps get an explanation about people’s actions towards our family?*

*That I do not bear children is also the doings of F., I have already filed charges and asked for the protection from my husband, which hasn’t been granted, since I have forcibly renounced it when giving testimony and he is needed for the satisfaction of Windhukers’ gossip.*

*‘Oh women, what a dirty butcher you have in your bed’!*

*I only would like to know what this war of men is for.*

*In reverence,*

*I.L.*

*I.F.*

## Locating I.L.’s Narrative in German South West Africa

In bearing empathic witness to I.L’s words one would need to be cautious not to attempt to reveal the truth, or a truth, about her account. One would never know if her allegations were true, or if those who raised the allegations against her were right. It is even impossible to know whether I.L. was mentally ill. I.L. herself is full of doubts and often appears confused. She makes her case forcefully—she demands government’s assistance, financial support and the opportunity to be repatriated—but she also pleads with her interlocutors to make sense of the accusations against her, of the gossip and the exclusion she had to endure. She wants to understand the reasons behind people’s conduct. Whilst anthropology has laid claim to be capable of making sense of other people’s experiences (Wagner 1981), psychiatry has argued that its foundations lie in restoring the patient’s delusional talks to reality (Das 2016). My aim here is neither to make sense of I.L’s story nor to ground it in reality. Instead I wish to locate her narrative within the context of the colonial world. It is her words that are revealing of the colonial world. It is her story that turns into an historical account. In so doing I hope to maintain I.L.’s authorship and avoid falling into the trap of what some have called ‘ethnographic ventriloquism’, the claim, as Geertz put it “To speak not just about another form of life, but to speak from within it” (Geertz 1988:143).

Thus my aim is to follow I.L’s lead into the colonial world. A close reading of her letters show how these are interspersed with statements that reveal much about the dominant ideas of class, gender and sexuality in the colony. Statements such as, ‘It is my language that is placed as common’, ‘I should be called a prostitute’, ‘Without wearing a ring I am not allowed to enter the cooperative’, ‘I came to DSW as a Government bride’, ‘he obtains no reputation from me’, ‘of what concern is to the public at what age I marry?’, and so on, reveal very important aspects of life in the colony. In the next sections, I will explore the relationship between class, gender and sexuality and how these intersects with racial discourses and practices to reveal the vulnerable position that working class women settlers occupied in German South West Africa.

## The ‘Woman Question’ in the German Colonies: Culture, Race and Identity in German South West Africa

*The German soldier conquers the land with the sword, the German farmer and businessman searches for its economic utilization, but the German woman is alone called and capable of keeping it German*,

Adda von Liliencron, Die Frauenfrage in Den Kolonien, Kolonie und Heimat, 1908-*09*^17^

Women occupied a particular space in the German colonial project (Carstens and Vallhorsts 2002; Bechhaus-Gerst and Leutner 2009; Walther 2004; Walgenbach 2005). In the imperial ideology of the Second Reich, women were assigned a crucial role to fulfil the dream of building of a ‘New Germany on African Soil’ (Walther 2002). Women were central to the moral and political economies of production and reproduction in the colonies. They were portrayed as the bearers and carriers of German culture, *Kulturträgerin*, and the keepers of Germanness, *Deutschtum* (Wallace 2011:194). In the ideal colony, women would bear children and establish proper German families; they would act as the buffer between German men and African women; and help maintain the racial purity of settler society against the fear of miscegenation and racial impurity (Conrad 2011; Lindner 2009; Švihranová 2014). Women thus occupied a central space in the creation and maintenance of the boundary between the colonizers and the colonised essential for the preservation of the binary racial order.

The ideal colonial world of racial and cultural purity required an immense financial and ideological effort on the part of the colonial government. It also required, as I will show, new legislations and new forms of control and coercion of both European settlers and Africans (Wallace 2011; Hartmann 2007a). Aristocratic and bourgeois middle class German women helped fuel this ideology through publications, in both the popular press and in the literature, and the establishment of women’s associations. Women such as Frieda von Bülow, Adda von Liliecron and Clara Brockmann and the colonial women organisations they established and promoted, the *Frauenbund* and the *Deutschen Frauenverein für Krankenpflege in den Kolonie* (The Association of Nursing in the Colonies) were central to the imperialist project of the Second Reich (Wildenthal 1993, 2001).

The popular press at home and in the colonies, idealised the image of women as *Kulturträgerin*. In a letter published in the *Swakopmunder Zeitung,* on March the 8th 1908, the president of the newly established *Frauenbund*, Baroness von Liliencron, encouraged the readers to disseminate the aims and objectives of the *Frauenbund*. Von Liliencron stressed that the role of the women’s league was to assist the successful integration of single women and girls into the colonies, through activities such as education, housekeeping, and nursing. Similarly regular articles published in *Kolonie und Heimat*, the magazine of the *Frauenbund*, stressed the importance of women’s civilising mission. The press also helped in creating an idealised image of the lives of German women settlers in the colonies, as well as providing important information on body care and women’s health, on shipping services to the colonies and the work of the *Deutsche Gesellschaft fur Kolonisation*, the German Colonial Society.

The debate on the ‘woman question’ in the colonies was central to public debates in both metropolitan and colonial societies. Between 1908 and 1913 several editorials and articles appeared in the colony’s major newspapers, *Winduker Nachrichten, Swakopmunder Zeitung and Lüderitzbuchter Zeitung*. Often the contributors to these topical debates and their interlocutors resided in Germany and the colonies. For example, in 1910 an epistolary appeared in the *Winduker Nachrichten*. In an impassionate discussion on the Frauenbund and the ‘woman’s question’, Pastor Hasenkamp of the Rhenish Mission in Swakopmund, and Dr. Philaletes Kuhn, former medical officer in the Nama/Herero war and now health advisor for German South West Africa in Berlin, engaged the readership with their opinions. In responding to Hasenkamp’s initial letter, Kuhn reminded him that the objectives of the *Frauenbund* were clearly stipulated in their original constitution,

1) To interest women of all class (es) in colonial issues; 2) To assist women and girls who want to settle in the colonies and to encourage women’s migration to the colonies; 3) To promote the school question in the colonies; 4) To assist women and children, and the needy; 5) To foster and strengthen the economic and spiritual connection of women in the colony with the homeland. Windhuker Nachrichten 29/10/1910^18^

Despite the *Frauenbund*’s original intention to promote colonial issues across all classes, class discourses permeated every aspect of the *Frauenbund*’s work. As Wildenthal (1998) argues, while many women adhered with ardent zeal- wanted to bring civilization to Africa, continue the German expansionist policies and defend the superiority of the German race- the nature and extent of women involvement in the colonial project remained ambiguous and fractured along class distinctions. Whereas the aristocracy and the upper classes found new freedom in the colonial enterprise and its spirit of adventure, working class women’s experience of the colonial world, like I.L.’s, remained limited within the confines set by sharp gender and class distinctions.

## Colonial Literature, Class and the Predicaments of Gender

This ambivalence emerges more clearly in the writings of von Bülow, von Liliencron, Clara Brockman and Lene Haase. Women, these authors argued, must be adventurous and independent, whilst remaining subservient to men’s authority, all in the interest of the Imperial colonial project. This message resonated particularly with middle class female audiences who were beginning to enter the public sphere of German society. Bourgeois colonial authors projected this image of emancipation and middle class aspiration by suggesting that women could do the same tasks as men. They could ride, shoot, and in the process civilise the African continent. And they could and must do so, while retaining their femininity. While men had conquered the land, through war and valour, they could not finish the job without women’s help (Bowersox 2013). However, the message remained ambivalent: while women were encouraged to seek adventure in the colonies, their role remained confined instrumentally to the domestic sphere. It is there that their work would be truly invaluable for the colonial project. In the home, they would be training and disciplining female workers who had begun to enter European settlers’ homes after to the serious shortage of labour that followed the genocidal Herero/Nama war of 1904/1908 (Hartmann 2007a). The colony needed strong, adventurous and domesticated women capable of producing and reproducing for the colony and the empire.

This gendered colonial ideology is best exemplified in Brockmann’s essay ‘*Die Deutsche Frau in Südwest*-*Afrika* (The German woman in South West Africa) (1910) and *Du Heiliges Land* (You Holy Country) (1914), and in Lene Haase’s novel ‘*Raggys Fahrt nach Südwest’* (Raggy Travels to the South West) (1910). The novels, set in the context of the diamond rush of the first decade of the twentieth century, are autobiographical in style and content. The novel’s heroines, like the authors have travelled to the colony. And like the authors, they are middle class, independent, educated and adventurous. They ride horses and can use a rifle. However, in these novels the heroines, despite their independence, could truly be ‘free’ with a man at their side. In commenting on Lene Haase’s novel Gudrun Thiel observes, “The social order is perpetuated when Raggy marries within her own class and accepts social responsibilities” (1988:46). Ultimately these colonial novels embody middle class aspirations of women’s emancipation but in an unchanged patriarchal world.

Colonial novels played a very substantial part in promoting the ideas behind “Imperial Feminism”, the ideology that encouraged women to take an active role in the public sphere and to contribute to the empire with their work and sacrifice whilst promoting and defending racial purity (Wildenthal 1998). Women like Frieda von Bülow, supported the use of violence, and racism in the colonies as necessary to respond to the threats of miscegenation. “Imperial Feminism” fed on social Darwinism by promoting the emancipation of racially superior German women. Racial and class order required a clear hierarchy of male power and female subordination, and in turn between white and black women. As Wildenthal argues, ‘Freedom of white women, ultimately was possible through the subordination of black men and the degradation of black women’ (1998:71). In this sense the most fundamental need of a woman was not freedom but subordination to the patriarchal world and to the colonial ideology of racial domination. As I will show in the next sections, the colonial novels and the work of the *Frauenbund* provided the ideological and material support to the process of building a German colony after the Nama/Herero genocide. This process was going to transform the moral and political economies of the colony.

## Gender, Race and Colonial Intimacies

In the project of rebuilding the colony after the Herero/Nama war, women would play a central role. Encouraged by imperial propaganda and the dream of wealth and independence, several thousand women, settled in the colony between 1908 and 1913. While the arrival of German women was triumphantly celebrated in the popular press, their appearance was received very differently by the colonial society and by men in particular. As Hartmann underlines ‘The direct attempts of the *Deutsche Kolonialgesellschaft* to provide women for marriage to German colonial men met reservations’ (2007a:67–68).

In this context single women, like I.L., occupied a very weak place in the colonial hierarchy. Numerically outnumbered three to one by men (Henderson 1962), German women were vulnerable.^19^ In post-genocide German South West Africa, women remained restricted within the home as home-makers and wives, protecting the household, and by extension the colony, from the sexual and racial dangers posed by African women. While Brockmann in her writings comfortably evades the issue of German men and their sexual conduct, it was women, she argued, who had to maintain and preserve the racial purity as opposed to men’s presumed weaknesses. Women had to fulfil the patriotic duty of carriers of German culture (Walgenbach 2005), preserving Germanness and racial purity, whilst contributing to the economy of German South West Africa. Women in German South West Africa came to embody the moral and political economies of the colony.

The *Frauenbund* provided the ideological and material foundation for the colonial project. By following rigid immigration laws, the *Frauenbund* selected the applicants for the colonies. Each candidate provided a set of documents to support their application. These included one photo, one medical certificate, a police report of good conduct, one certified personal document, and a letter of acceptance of employment.^20^ These rigorous procedures and checks were put in place to stop ‘undesirables’ entering the colony. Furthermore, the employment contract that women signed through the *Frauenbund* was particularly draconian. It was a contract that had no return ticket. The onus was left on the women to find a man, to marry, have children and settle in the colony for good. It was specifically stated that unmarried women, who were encouraged to apply to receive the *Frauenbund*’s aid, were ‘strong and healthy girls who come from the country, have considerable experience of domestic work, and are unpretentious in the manner of living’^21^ Furthermore unmarried applicants would need to state that their father or legal guardian approved their decision to go to the colony (Pierard 1971:319).

Most of the applicants were working class women. They entered contracts of servitude and their hopes to advance in status in the colonial hierarchy was limited to that of married woman and mother. And despite the *Frauenbun*d’s bourgeois aspirations to transform and emancipate these women, their position in the colony remained unchanged (Wildenthal 2001). Women were needed to produce and most importantly reproduce enough children to strengthen numerically settler society and to stop miscegenation. By civilising African women, and teaching them how to work in the domestic sphere, women would educate the natives to obey their masters. In this context, settlers’ households became ‘intimate sites of colonial implementation’ (Stoler 2002).

However, I want to argue, that in sharing the domestic sphere with African servants, European women were brought into new spheres of intimacy, which revealed not only the production and reproduction of the colonial world, but also novel spaces of vulnerability. With both confined to the households, albeit in different hierarchical positions, German women’s bodies became interchangeable, argues Wildenthal (2001), with that of African women, as both became the objects of men’s sexual desires. In these novel spaces of domesticity and shared vulnerability, working class white women were only one step away from the threat of colonial violence, and the possibility of falling into the category of the ‘undesirable others’.

In fact, alongside the fear of Europeans going native, and that of miscegenation, the poor, marginal and underprivileged whites like I.L became a threat to ‘*La Situation Coloniale*’ (Balandier 1951). As Conrad (2011) underlines the intersection of class and gender with racial ideologies created ambiguous and competing positionalities that left many white settlers, especially poor, unskilled working-class men and women very vulnerable to the colonial order.^22^

The project of building an ideal colonial society, based on classless distinctions and with clear racial boundaries, remained a mere fantasy from the very beginning (Kundrus 2003). While the colonial government in Germany maintained the image of the idyllic colony, things on the ground were very different. Life in German South West Africa was very hard (Wallace 2011:196). The idea of the self-sufficient farmer, transforming the land and living off it, remained a mere dream. Farm ownership also was very limited, especially for women, who had little or no access to land (Wallace 2011). As Wallace underlines, the government efforts to control the behaviour of white settlers, ‘illustrates both the difficulties of creating and sustaining whiteness and Germanness as dominant racial categories, and the extent of class tensions in the colonies’ (Wallace 2011:196).

## Gossip, Rumours and the Desirable/Undesirable ‘Others’

The colonial government tried with great efforts to eliminate the threat of white poverty from German South West Africa and its other colonies. In this process of cleansing new categories of undesirables emerged: alcoholics, indigents, the mentally ill, syphilitics and homosexuals. Anyone falling in these categories could face deportation to Germany. As Schmidt (2008) argues, the idea of propriety and morality remained central to the colonial projects. Gossip and rumours were central to the life of the small colonial societies, regulating social relationship and maintaining class, gender and racial distinctions.

Gossip, rumours and scandals were central to maintain the social order (Gluckman 1963). Colonial society, as Schmidt suggests, rested not only on the clear boundaries between Europeans and Africans, but also on the moral conducts of European settlers. Writing about the famous Rechenberg scandal in German East Africa for example, Schmidt argues that the scandal showed the fragility of colonial society and its construct of the unchallenged superiority and legitimacy of colonisers. “The scandal”, Schmidt writes, “Showed how closely colonisers and colonised shared the same space to the point that the colonial society stood revealed if not naked in front of colonial servants and its employees” (2008:33–34).

The attempts to maintain control over the intimacy and sexuality of both European settlers and the African colonial subjects, left working class women extremely vulnerable. Saddled with many tasks, ‘she [the German woman] was supposed to cultivate the community, to take care of her families health, to prevent man’s alcoholism and, last but not least, to prevent a German man from short-term affairs or long-term relationships with native women.’ (Švihranová 2014:300). German women inevitably failed to fulfil these duties, in particular stopping German men from establishing liaisons and relationships with African women. And while upper-class German women maintained more or less successfully their racial distance from their position of privilege, and continued to idealise in their writings the role of the German woman as independent and emancipated, working class women drawn into novel spaces of shared domesticity with African women became increasingly disempowered (Hartmann 2007b).

After the war, the shortage of labour encouraged an increasing number of African women to move to the urban areas of South and Central Namibia, in particular to Windhoek and Swakopmund (Hartmann 2007b). Whilst in the past African men had also worked in European households, African women were now needed to do most jobs including working as servants. Brought into these domestic spheres they established intimate liaisons with their male employers. The nature of these intimate relationships need of course to be inscribed in what Hartmann (2007b) and O’Donnell (1999) describe as a context of sexual coercion, violence against women and prostitution. As Hartmann (2007b) shows in his work, prostitution in the colony, formal and informal, was by this time an important feature of its social geography of everyday life.

During, and especially after the war, however, with the rise and affirmation of racial theories of miscegenation and the threat of venereal diseases, white prostitution became formalised (Warmbold 2010). Also, the intervention of the missionaries, who saw the colony slumping towards degeneration (O’Donnell 2005), and the role of biomedical discourses on venereal diseases contributed significantly to this formalisation (Wallace 2002). According to Hartmann, in Windhoek alone there were fifteen brothels hosting exclusively European women,^23^ ‘But now (after the war) men preferred the services of white prostitutes because they were much more regularly checked for venereal disease’ (2007b: 51).

It is in this context of violence, the fear of degeneracy and racial mixing, and the new shared intimacies between Europeans and natives developing in the colonial household, that moral panics and scandals reverberated across colonial society. Rumours of poisoning and of employees attacking their employers circulated widely in the colony. In the rising clamour of colonial paranoia, male and female employers resorted to brutal violence to punish their employees, leading in some well-known cases to murder.^24^ As O’Donnell points out (1999), just like rumours and gossips, these narratives reveal not only racial and sexual anxieties, but also social tensions fuelled by colonial paranoia. These conspiracies and the violence that accompanied them reveal the colonial anxieties about colonial production and reproduction, as well as the nature of patriarchal and racial relations in the colonies. They also bring to the fore how in the shared spaces of intimacy of the colonial world, especially in the domestic spheres, white working class women occupied unexpected positions of vulnerability in the brutal and violent context of German South West Africa.

And it is in this context that one needs to place I.L’s words and her desperate plea for help and the recognition of her humanity.

## Conclusion: Bearing Witness to I.L

We left I.L. on the 4th of April 1916. After her second letter, a third and shorter note followed, the correspondence between the German authorities and the South African military administration intensified. The German authorities requested I.L.’s immediate confinement to an asylum. To this end they offered to pay for I.L.’s transportation to the Union of South Africa. German South West Africa did not have an asylum and in previous cases, the mentally ill had been repatriated to Germany in the care of relatives and/or to the confinement of an asylum.^25^ While the South African military administration agreed to this solution there were issues of jurisdiction over German subjects, de facto prisoners of war, which needed to be carefully considered. In particular, what legislative framework should they use- the German Imperial code or the Union of South Africa’s laws?

The South African administration extended the 1897 Lunacy Act of the Cape Colony to South West Africa.^26^ The act required two independent medical reports to authorise the confinement in an asylum. Dr Kannegisser, a local physician who had opened his practice in Windhoek in 1911,^27^ provided the first report. In it he describes I.L. as a dangerous lunatic ‘suffering from Paranoia, an illness, which is connected with preconceived ideas and hallucinations.’ Dr Kannegisser called for I.L’s immediate confinement in Valkenberg.^28^

However, with only one report, I.L. refused to see Dr Brenner for the second assessment, the South African administration asked the local municipality to produce in its stead three ‘certificates’ from people known to I.L. who could testify to I.L’s insanity. At the request of the German municipality three witnesses provided a statement. These were mailed on May the 5th 1916 by Mr. Junker, the deputy Mayor of Windhoek to Captain Overbeck, the military magistrate in Windhoek.^29^ While both the municipal accountant Mr. Gruschka, and the Windhoek deputy mayor and bank director, Mr. Junker, simply confirmed I.L.’s infirmity, ‘Her expressions and talks when she speaks and writes are of a kind, which is possible merely from a person whose mind is not clear’, the third ‘certificate’, is perhaps the one that reveals much about Windhoek colonial society and the place of women in it. This is the statement of Mrs Kriele, the president of the local *Frauenbund*,

*On your question I beg to reply that I know the lunatic I.L. since the time she came to this country. In the earlier days she used to come to the girls’ evenings, which were held by me in the parsonage. Also at other times she used to visit us until she left for Swakopmund, and she wrote numerous letters to us. We at once perceived from what she said and wrote that I.L. is completely mentally insane. She is suffering from hallucinations, she hears everywhere voices and by this she always expresses her fear lest somebody is threatening her life. By these delusions she invents the most mean [sic.] stories and she wrote whole treatises of this kind. Therefore my husband and I are of the opinion that in her own interests and with regard to the other people, I.L. ought to be transferred to an asylum as soon as possible*.^30^

Feminist scholars have long argued that the marginality and powerlessness of women is reflected in both the ways women are expected to speak, and the ways in which women are spoken of (Cameron 1995, 1998). Women’s speech, feminists argue, is located within power differentials determined by gender, race and class. When women speak their language is weighed always in relation to the patriarchal world they inhabit and which limit their capacity for self-expression and language competence within what is expected of them, in relation to their positionality in the social world. Mrs. Kriele’s words, as a prominent member of Windhoek’s colonial elite and the president of the *Frauenbund*, would have undoubtedly carried a lot of symbolic weight, and perhaps contributed significantly to the decision to commit I.L. to the Valkenberg Asylum on July the 18th 1916.^31^ But perhaps, in some sense, Mrs Kriele also vindicated I.L, as her letter exposed a social world in which class and gender shaped the experiences of European settlers and Africans alike.

In bearing empathic witness to I.L, I rested my argument on the premise that one should take I.L.’s words seriously, not because they reveal a truth about her condition, but because of their capacity to reveal her subjectivity, her shifts in the efforts at coherence and self-making (Fumanti 2018). Her words aimed to reverse the sense of ‘social defeat’ that she had experienced and in so doing recover her shattered biography through her effort at autobiography. Paradoxically, one could say, her desperate efforts accelerated her confinement to the asylum, as her words were perceived as the evidence of a deranged mind. Here, I agree with Gilles Deleuze who, in his analysis of the condition of delirium, captures the paradoxical aspect of this phenomenon, ‘I think that the delirium has great potency. The delirium in and of itself has great potency. But it is the person that raves that can be reduced to impotency’ (Deleuze in Stivale 2011).

So when I say I took I.L. seriously, I did not intend to emphasise her words as the clear manifestation of her diagnosed psychosis, or the revelation of an absolute truth about her life or that of others. On the contrary, I suggested that her words revealed, in her efforts to escape medicalization and institutionalisation, important aspects of the moral and political economies of German South West Africa. In this sense I.L’s story is both a story and an historical account. It is an historical account because it helps understand the relationship between power, gender, class and race in the colonial world. It also reveals important insights into the mental illness narratives and experiences of European settlers in German South West Africa and more widely in colonial Africa.

## Footnotes

1 Arnold Schad served as mayor of Swakopmund between 1915 and 1918, and between 1920 and 1931.

2 NAN, SVW, G.5.AE.

3 NAN, ZBU, 1911, BWI/208.

4 There is now an established scholarship that examines patients’ letters in Britain’s asylums (Barfoot and Beveridge 1990; Beveridge 1997, 1998; Smith 2008).

5 For a comparative analysis with Oceania see Coleborne (2015).

6 NAN, SVW, G.5.AE.

7 NAN, ZBU, Kaiserliche Bezirksamt Windhoek, Akten B.10.N, Band II.

8 NAN, SVW, G.5.AE.

9 NAN, SVW, G.5.AE. In a note to a letter from Mr Grumpecht, the Swakopmund notary, Mr Bult, the military magistrate, explains that the Red Cross ‘Deals with all questions of travelling, expenses, hospital fees of German indigents. The English Government only deals with the question of rations’.

10 The Woermann Linie provided passenger and shipping transport between Hamburg, Swakopmund and Lüderitz. In 1916 the company was sold to the Deutsche-Ost Afrika Linie.

11 I am aware of the complex issues of translation and representation in archival research (Munday 2013). I tried to remain as close as possible to the original text with the assistance of an expert translator, Lisa Levi.

12 I.L.’s gets Kastl’s name and position wrong. Under South African martial law, Kastl was the leader of the interim German administration and acting commissioner for the mandate power.

13 NAN, SVW, G.5.AE. Hans Uhlemann was the owner of the Hotel Hohenzollern in Windhoek.

14 Dr Stark was a lawyer and prominent member of colonial society.

15 Victor Franke was a military officer and the last commander of the Schutztruppe.

16 Dr Brenner was the registered physician in Swakopmund’s district hospital.

17 *Kolonie und Heimat*, 4, (1908), p. 8.

18 *Windhuker Nachricthen*, National Library of Namibia.

19 In 1914 out of 14,806 settlers nearly 3000 were women.

20 NAN, ZBU, 1911, BWI/208.

21 Deutsch-Südwestafrika. Amtlicher Ratgeber fur Auswanderer (Berlin 1912).

22 In this position of vulnerability, European settlers often resisted deportation. NAN, ZBU H. II, i. 3.

23 On the emergence of a global prostitution racket across Southern Africa, see Charles van Onselen (2007).

24 The most famous case is that of Ludwig Cramer, a German farmer in the Gobabis district whose brutally mistreated and killed his workers.

25 NAN ZBU H.II, i. 3, Gesundheit Polizei, Geistekranke Weiss, Specialia.

26 Until the introduction in 1916 of the Mental Disorders Acts 207 different legislations, for the detention and treatment of ‘mentally disordered and defective persons’, were in force across the provinces of the Union of South Africa.

27 NAN, ZBU, B.III, v2.

28 NAN, SVW, G.5.AE, Dr. Kannegisser, Letter to Municipality, 26/04/1916.

29 NAN, SVW, G.5.AE, Mr. Juncker, Letter to Military Magistrate, 05/05/1916.

30 NAN, SVW, G.5.AE, Mrs. Kriele, Certificate to Municipality, 05/05/1916.

31 NAN, SVW, G.5.AE, Secretary of the Protectorate to the Mayor of Windhoek, 22/07/1916, for a study of Colonial Valkenberg see Marks (1999).
